# The visual story of data storage: From storage properties to user interfaces

**DOI:** 10.1016/j.csbj.2021.08.031

**Published:** 2021-08-24

**Authors:** Aleksandar Anžel, Dominik Heider, Georges Hattab

**Affiliations:** University of Marburg, Department of Mathematics and Computer Science, Marburg 35043, Germany

**Keywords:** Storage, Device, Medium, Usage, Capacity, Lifespan

## Abstract

About fifty times more data has been created than there are stars in the observable universe. Current trends in data creation and consumption mean that the devices and storage media we use will require more physical space. Novel data storage media such as DNA are considered a viable alternative. Yet, the introduction of new storage technologies should be accompanied by an evaluation of user requirements. To assess such needs, we designed and conducted a survey to rank different storage properties adapted for visualization. That is, accessibility, capacity, usage, mutability, lifespan, addressability, and typology. Withal, we reported different storage devices over time while ranking them by their properties. Our results indicated a timeline of three distinct periods: magnetic, optical and electronic, and alternative media. Moreover, by investigating user interfaces across different operating systems, we observed a predominant presence of bar charts and tree maps for the usage of a medium and its file directory hierarchy, respectively. Taken together with the results of our survey, this allowed us to create a customized user interface that includes data visualizations that can be toggled for both user groups: Experts and Public.

## Graphical abstract

**Figure:**
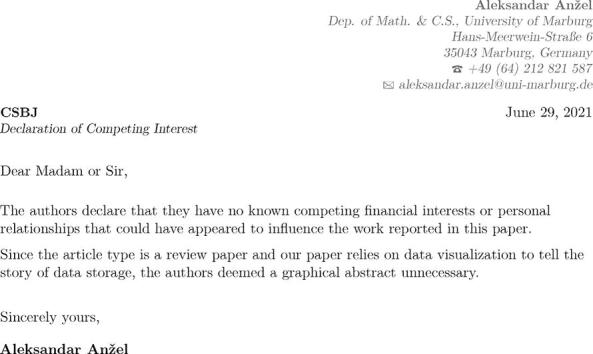

## 1 Introduction

At our current rate, 2.5 quintillion bytes of human and machine-generated data are created every day, and the pace is only accelerating [1]. The amount of created data in 2020 was predicted to reach 35 zettabytes or ZB (i.e., 35 trillion gigabytes or GB). 33 ZB were already reached back in 2018. This lead the International Data Corporation (IDC) to make a new estimation for 2025: 180 ZB of new data will be created worldwide [2]. Although such numbers are not astronomically large, they remain too large to fathom. For comparison, the radius of the observable universe is roughly $43\times 10^{21}$ kilometers; which is two orders of magnitude smaller than the aforementioned estimate of 180 ZB or $1.8\times 10^{23}$ bytes [3]. With the advent of such a historical era, better storage devices and long-term storage solutions will be required. This especially holds true with a projection putting the world population around 10.88 billion in 2100 [4].

A storage device can contain information, process such information, or do both [5]. When the device contains only information, it is called a recording medium. Recording can be done using almost any form of energy, acoustic vibrations (phonographic recording) to electromagnetic energy (magnetic tape and optical discs) [6], [7], [8]. Today’s storage devices contain different types of magnetized media that are usually ferromagnetic materials (e.g., ion or chromium oxides) [9], [10]. Ferromagnetic materials have structurally unpaired spins that are organized into magnetic domains. Many bytes, as the units of information, can be recorded on magnetized media as ”ascending” or ”descending” spin domains. These correspond to ones and zeros in the binary system. Thanks to the unique property of ferromagnetic materials, these magnetized media retain the data, and they can then be read in the same way. To provide a descriptive overview of storage devices and media, we follow their basic properties: (1) Accessibility, defines how data is organized on a device and how it can be accessed: serial or random [11]; (2) Capacity, defines how much capacity a device has (in bytes) [12], (3) Lifespan, defines how long data can be stored in certain conditions (in years) [13], [14], (4) Mutability, defines the functions of a device: write, read, or both; and (5) Typology, defines the categories of storage devices: optical, magnetic, semiconductor or electronic, molecular, etc. Combined with other properties, such as energy use or data density, they affect the adoption and usage costs of a specific device or medium. For example, data density is a measure of the quantity of information bits that can be stored on a given length of track, area of surface, or in a given volume of a storage medium. A higher density is preferred to optimize the given length, surface, or volume of said medium. Altogether, such properties steer the adoption of storage devices and media not only by commercial companies but also by the public. In the context of long-term data storage, devices with volatile memory are faster than non-volatile memory. However, in the event of a power outage, volatile memory is not retained rendering it unsuitable. In the looming possibility of a digital dark age, this rationale extends to digital libraries and renders them obsolete, e.g., tapes or network storage systems (clouds). This, in turn, led to the development of alternative and novel media.

Striking examples of novel storage media are Ribonucleic acid (RNA) and Deoxyribonucleic acid (DNA) molecules. Because of its greater stability, the DNA molecule presents a better storage medium than its counterpart RNA. It carries the biological information necessary for the proper functioning of cells [15], [16], [17]. It is not by any means new, as it has been used for billions of years as a carrier for genetic information by living organisms [18]. Compared to other – organic and inorganic – molecules, its capacity puts it in a league of its own [19], [20]. DNA has a great potential for information storage with a capacity outperforming existing technologies. For instance, traditional storage devices such as magnetic hard drives and flash drives have a data density of $10^{13}$ and $10^{16}$ bits per cm^3^, respectively [21]. In comparison, DNA reaches a data density up to $10^{19}$ bits per cm^3^. In other words, three orders of magnitude higher and specifically of 1 billion terabyte per gram. Moreover, DNA has a very good molecular stability, which has been shown in sequencing studies of extinct species, referred to as ancient DNA [22], [23].

Aside from their novelty, a yet-to-be-considered aspect concerns the User Interface (UI), which of the aforementioned properties are relevant, and how they are visualized. For instance, knowing if a Hard Disk Drive or HDD device is at 20% or 50% of its usage is often visualized using a horizontal stacked bar chart. However, with the advent of new storage media, such as molecular media, there exist no known standards. That is to say, there is no agreement on which properties are more important or relevant for a potential user. Indeed, user preferences impact which information is judged as relevant, then subsequently visualized. In this work, we argue that certain standards should be considered to prepare for the foreseeable future where such media may be available for the task of long-term archiving or even for day-to-day use. For this purpose, we investigated the industry-wide approaches that implement UIs and visualizations to display some of these storage properties. We reported a historical account of their evolution across operating systems and platforms. We also divided the timeline into three distinct periods (magnetic, optical and electronic, alternative) and reported the TOP 3 media and devices for each time period. To accommodate for the shift of storage types into molecular media, we created a survey to rank the storage properties by importance depending on whether the user is a member of the general public or the research community (i.e., domain expert). The expert pool consisted of members of the largest known research consortium on MOlecular Storage for Long-term Archiving (MOSLA). We summarize our findings below:

(a) We conducted a successful literature search of historical, currently in use, novel and experimental data storage media and devices,
(b) we created a survey to determine the most important storage media properties depending on a target group, and
(c) by relying on our findings, we proposed a user-settable UI to select and display the relevant properties for the right audience.

## 2 Results

We report the results in five subsections. First, we focus on presenting specific storage media and devices that relate to the typology, accessibility, and mutability properties. We rely on the typology property to delineate periods of time for making the timeline more tractable. Second, we present storage media and devices by capacity and lifespan. In each subsection, only the TOP 3 is reported for the sake of brevity. Third, we briefly describe novel and alternative media as they bring in their own right a new set of challenges. Fourth, we report the results of our survey on user preferences to visualize storage properties. Fifth, we detail our proposed UI that can be toggled depending on the target audience with historically adapted visualizations and user-settable parameters.

### 2.1 Timeline by typology, accessibility, and mutability

First, we report the timeline of different storage media by their type. Second, and for brevity, we consider the logical condition of reporting the TOP 3 storage devices and media that are only and only if both randomly accessible and mutable (read and write).

**Fig. 1:**
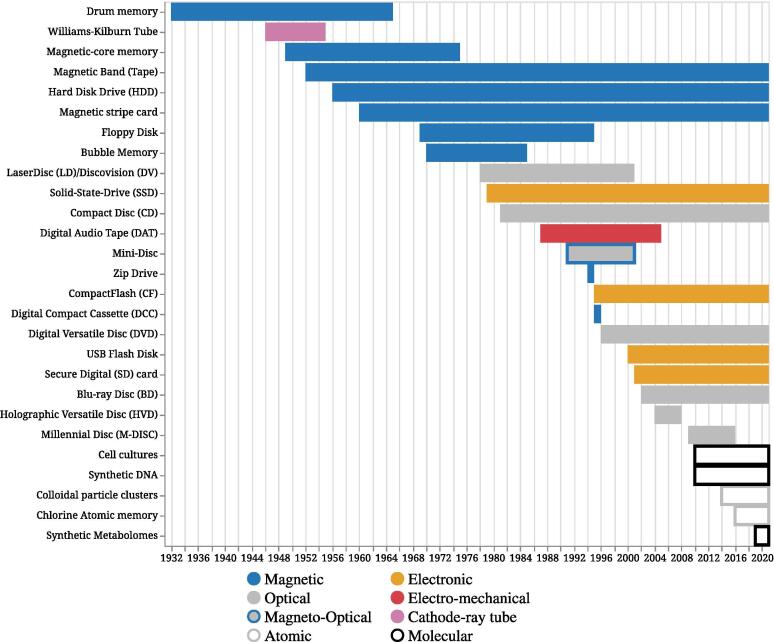
Timeline of Storage Media and their Usage. The transition to new storage technologies is observable at multiple time points. Today, various storage media from different eras are still in use.

**Fig. 2:**
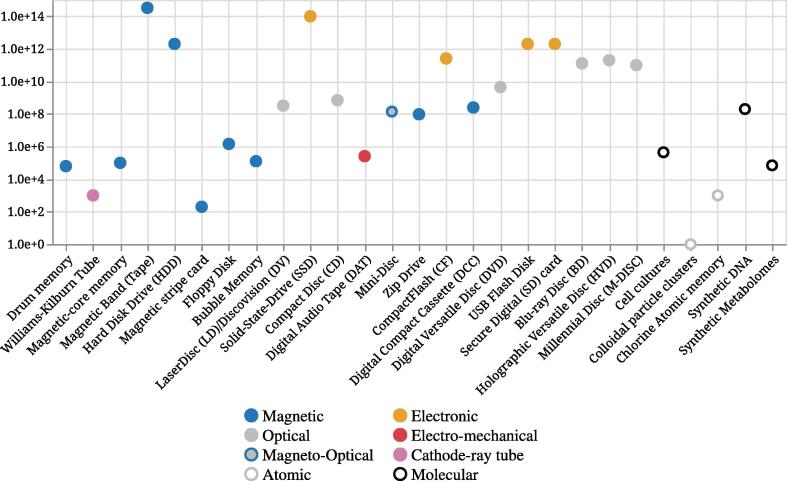
The Capacity of Storage Media over Time. Values in bytes are reported on a Log scale with a base 10. It is possible to observe an increase over time as novel media improve in capacity. However, new experimental devices or media are often expensive which limits the storage capacity.

In the beginning, data storage heavily relied on magnetic media. As seen in Table 1 and Fig. 1, Fig. 2, the magnetic time period is observed in blue. This magnetic era slowly shifted to optical and electronic media (in orange and gray). In addition, experimental storage technologies with hybrid typologies saw the light. For example, electro-mechanical and magneto-optical typologies, DAT (in red) and Mini-Disc (in gray and blue), respectively. This optical and electronic era continues to supply remarkable data storage and media. However, in most recent years, we have seen the rise of the novel and alternative media that effectively rely on atoms and molecules, while continuing to make extensive use of previous media types. Hence, the timeline of data storage devices and media can be divided into three time periods or eras.

**Table 1:** *Timeline of data storage media.* In the beginning, data storage heavily relied on magnetic media. This slowly shifted to optical and electronic media. In recent years, we have seen the rise of novel media that rely on atomic properties, biological molecules, and organisms. The magnetic Band (Tape) storage medium is reported in 1952, although BASF introduced the first magnetic tape for audio in 1934. Although BASF supplied the first 50,000 meters of magnetic audiotape in 1932, the magnetic tape is reported in 1952 as it corresponds to the first usage of a tape as a data carrier using the IBM 7 track. Mutability refers to read, write, or both. Note that there exists no device with a write-only mutability. We refer to the binary state of read and write (using the letter x) versus the read-only state [18], [24], [25], [26], [27], [28], [29], [30], [31], [32], [33], [34], [35], [36], [37], [38], [39], [40], [41], [42], [43], [44], [45], [46], [47], [48], [49], [50], [51], [52], [53], [54], [55], [56], [57], [58], [59], [60], [61], [62], [63], [64], [65], [66], [67], [68], [69], [70], [71], [72], [73], [74], [75], [76].

The first is the magnetic era, where the arrival of magnetic core memory and Hard Disk Drives revolutionized data storage as we know it. This era spanned ~40 years of almost exclusively magnetic devices. From the introduction of drum memory in 1932 to the bubble memory in 1970, magnetic devices starring this era were the Magnetic-core memory, the Hard Disk Drive (HDD), and the Floppy Disk. ***Magnetic-core memory***, or ferrite-core memory, is an early form of computer memory. It uses small magnetic ceramic rings, namely the cores, to store information via the polarity of the magnetic field that they contain. In 1949, the earliest work on core memory was done by Shanghai-born American physicist An Wang, who created the Pulse Transfer Controlling Device. The name referred to the way the magnetic field of the cores could be used to control the switching of the current [77].

***A Hard Disk Drive*** (HDD) stores and retrieves digital data from a planar magnetic surface and relies on rigid rotating platters. The information is written to the disk by transmitting an electromagnetic flux through an antenna or write head that is very close to a magnetic material, which in turn changes its polarization due to the flux. The first computer with an HDD as standard was the IBM 350 Disk File, introduced in 1956 with the IBM 305 computer. ***The Floppy Disk*** is a data storage device that is composed of a circular piece of thin flexible magnetic medium encased in a square or rectangular plastic casing. A Floppy Disk is read and written using a floppy disk drive. In 1967, IBM started developing a practical and inexpensive device for easy loading of microcode into their 370 mainframes and for sending out customer updates. The result of this work was a read-only, 8-inch (20 cm) floppy. Initial floppy disks were designed to hold 80 KB and load microcodes into IBM 3330, making them an intermediate device to fill another storage device, i.e., a disk pack file with a 100 MB capacity. Next disks were 5.25 inches, then dimensions changed to 3.5 inches and with added protection thanks to a sliding metal cover to protect the disk medium from direct physical contact. With new floppy sizes and competitive prices, newer and smaller floppies replaced their predecessors very quickly.

The second is the optical and electronic era. Although other hybrid device and media types were introduced in this era, the optical and electronic types were the most prominent. It spanned 24 years, from 1978 to 2002, with many media and devices that were both accessible and mutable. It all started in 1978 with the LaserDisc. ***The LaserDisc*** (LD) is a home video format and the first commercial optical disc storage medium. In the same year, in 1978, StorageTek${}^{®}$ launched the STC 4305, which was the first semiconductor storage device compatible with a hard drive interface, or ***the Solid-State-Drive*** (SSD), aimed at the IBM mainframe plug-compatible market. It entered the market as a serious competitor to the IBM 2305 HDD system with seven times the speed and half its price. The LD was the predecessor of the Compact Disc (CD), the Digital Versatile Disc (DVD), and the Blu-ray Disc (BD) which were launched in 1981, 1995, and 2002, respectively. While the SSD was the predecessor of flash memory-based media, namely the Compact Flash (CF), the USB Flash Disk, and the Secure Digital (SD) card, which were launched in 1994, 2000, and 2001, respectively. Introduced in 1994, ***the Zip Drive*** failed to replace the widely adopted 3.5 inches floppy disks although it was the super-floppy of the era with a much greater capacity and performance.

The third is the molecular and atomic storage era. It spans from 2004 to today. Although this era features novel storage media, only Chlorine Atomic memory and Holographic Versatile Disk (HVD) are considered both accessible and mutable. From 2004 to 2008, ***the Holographic Versatile Disc*** (HVD) was researched and its development was halted. Compared to DVD technology, holographic memory could have stored information at higher density inside crystals or photopolymers [61]. In DVDs, the upper limit of the data density was reached due to the diffraction limit on the writing beams. Although promising, the HVD was never released. In 2006, ***Chlorine Atomic memory*** consists of arranging functional atoms into extended and scalable atomic circuits. The idea is to create an atomic-scale memory that can be read and rewritten automatically by means of atomic-scale markers using chlorine vacancies on a copper sheet (Cu) [75]. Such vacancies are found to be stable at temperatures up to 77 K (−196,15 °C) and would outperform state-of-the-art HDDs by three orders of magnitude.

Over time, storage devices improved in terms of accessibility. However, preferences for novel upcoming devices and media shifted their adoption and usage. As seen in Fig. 1, if the timeline is based solely on the use of storage devices, we observe a clear separation between storage devices that are still in use and those that are obsolete. For example, Floppy Disks became obsolete in the mid-1990s. From the magnetic era, accessible and mutable devices that survived only include HDDs. From the optical and electronic era, all aforementioned optical and electronic media are still being used today with the exception of the Blu-ray Drive which was discontinued in 2019, and later on, replaced with UHD Blu-ray which is now predominantly used. From the molecular and atomic storage era, Chlorine Atomic memory is the only accessible and mutable medium. Although this is the case, many other media have been developed with long-term archiving in mind. This led to a polarization of inaccessible and immutable media (e.g., synthetic DNA, synthetic metabolomes).

### 2.2 Timeline by typology, capacity, and lifespan

In this second subsection, we borrow the aforementioned division of the timeline and report the TOP 3 storage devices in terms of capacity and lifespan properties. Fig. 2 introduces the capacity of storage media over time on a Log scale.

First, magnetic storage devices proved to be resilient to the ever-growing need for more storage space. However, as electronic technology progressed, the capacity of magnetic-based storage devices became greater and the actual magnetic memory became cheaper. Because of their resilience and price, magnetic devices are widely adopted for archival use. ***Hard Disk Drives*** (HDDs) are the most developed magnetic-based storage medium. Their capacity increased substantially, from 3,750,000 bytes of IBM 350 disk storage unit to around $2\times 10^{12}$ bytes of modern HDDs [78]. The lifespan of any storage medium is directly influenced by environmental factors in which that medium resides, as well as the frequency in which it is used. It was long thought that higher temperatures might increase the chance of HDD failure, however, recent studies have found that there was no correlation between physical drive temperature and drive failures [79]. Since HDDs rely on mechanical parts for read and write operations, their lifespan is constrained to the quality and durability of those parts. New technologies of disk coating are being introduced to improve the current durability and data density of HDDs. Carbon-based overcoats are replaced with graphene-based ones to achieve a better reduction in friction and provide superior corrosion and wear resistance. This, in turn, enables the potential of increasing data density up to 4 to 10 times [34]. ***The Zip drive*** was the least long in use compared to all previously mentioned media and devices. Therefore, not much was done to further develop its capacity and lifespan. At the peak of their development, the maximum capacity of the Zip Drive was around $10^{8}$ bytes. The internal structure of the Zip Drive is almost identical to that of the HDD, consisting of read/write heads hovering over a rapidly spinning floppy disk mounted in a sturdy cartridge. That makes the Zip Drive much like the HDD, prone to failure and with a similar lifespan. ***Magnetic–core memory*** was introduced in the early days of computer systems and engineering. The properties of this storage device, such as non-volatility and random-accessibility, made it perfect for use as the primary memory of those early systems. The maximum capacity of the Magnetic-core memory was around $10^{6}$ but was not further improved, as the storage medium was replaced with a more technologically advanced static random-access memory.

Second, the optical and electronic era. With the introduction of the first optical storage medium in 1978, ***the LaserDisc*** (LD) provided a serious alternative for long-term data storage. Its introduction occurred roughly at the same time as the first electronic storage medium, the SSD. As previously mentioned, the LD preceded many optical media. ***The Blu-ray disc***, and its modern successor the Ultra HD (UHD) Blu-ray, currently holds the record for highest storage capacity for optical media of this era. When first introduced, the capacity of this storage medium was around $25\times 10^{9}$, while it now peaks at around $10^{11}$ bytes. Unlike the organic dye used in optical disc media, found in DVDs and CDs, a different approach is used to encode data on a Blu-ray disk. A combination of silicon and copper is used to create a layer on which the data is engraved, making Blu-ray disks significantly more durable and resilient than DVDs and CDs, with a lifespan of around 150 years. However, this warrants further research since these media did not exist long enough to either confirm or deny such claims. Alternatively, the current record for electronic media or storage devices of this era is currently held by ***the SSD*** with a maximum capacity of $10^{14}$ bytes. In second and third place, ***the USB flash disk*** and *the SD card* share the record with current maximum capacities of around $2\times 10^{12}$ bytes. Indeed, electronic-based storage devices do not contain any moving parts and therefore use entirely different methods to read, write, and store data. Research shows that the life span of devices based on flash technology is greatly determined by the usage in terms of data written on those storage media. Furthermore, it has been shown that SSDs were replaced 25% less often than HDDs [80].

The third era is represented by the introduction of many novel media such as cell cultures, synthetic DNA, and synthetic metabolomes. In addition, the Holographic Versatile Disc (HVD) optical medium was meant to compete with Blu-ray disk on capacity and reliability. However, HVD was costly and incompatible with existing or new storage standards, making its adoption problematic. The current storage standard specifies $2\times 10^{11}$ bytes of capacity. Yet with no clear demand, such disks were never put in mass production and the HVD remained in the research phase [81]. This third era is further detailed in the next section.

Over time and across media types, the changes in capacity reached in multiple instances local maximums. For example, optical media have reached a capacity of $10^{11}$, as seen in Fig. 2.

### 2.3 Novel and alternative media

Many novel and alternative media have been proposed in different contexts. They comprise atomic and molecular media. In the case of molecular media, a variety of approaches exist including organic, such as synthetic metabolomes, metallo-organic clusters, synthetic DNA molecules, and inorganic clusters. The most promising molecular medium is ***synthetic DNA***, although it requires a variety of steps; e.g., synthesis, encoding, sequencing, error correction, etc. Indeed, some approaches figured out that DNA storage systems are too slow to replace HDDs [64]. Yet more recent breakthroughs have made use of hundreds of thousands of short DNA oligonucleotides for encoding small amounts of data [65]. In that approach, naive encoding without error correction was used, which rendered it unsuitable for long-term archiving due to sequencing errors. To avoid problems in sequencing, a Huffman-encoding with a ternary code was employed by another approach [66]. This specifically helped avoid homo-polymeric DNA sequences which are problematic at the sequencing step. Moreover, they introduced redundancy in the encoding, thus enabling a simple error-correcting procedure. Based on these findings, another approach improved data density in the DNA storage medium by employing a kind of RAID (Redundant Array of Independent Disks) system [68]. In addition, a rudimentary random access procedure based on primer sequences was developed. Although it can be used for direct access of parts of the data, it is not possible to perform semantic searches, for which complex index structures are necessary [82]. For this reason, we did not report synthetic DNA as an accessible medium. Further research into improving DNA storage systems made use of different encodings (e.g., fountain codes [69], forward error correction [83], Reed-Solomon codes [67]). In spite of the fact that they are used for error correction, these encodings only correct or compensate until a certain threshold is reached. When the DNA storage molecule(s) are exposed to different reagents or stimuli, error-correcting codes cannot handle any potential degradation (e.g., damaged bases, breaks between individual nucleotides, and fractures in the phosphate backbone). The usage of higher codes such as Galois fields codes could help error- and erasure-correcting codes for reliable DNA storage [84], [85]. A comprehensive review of the research literature on synthetic DNA storage and its challenges is addressed by Dong et al. [86]. Physical storage of synthetic DNA varies from one laboratory to another. It defines a unit of molecular storage that often relies on sequencing redundancy (i.e., deep sequencing coverage, and having many copies of each sequence) and ranges from, but is not limited to, amber-enclosed spores [18], lyophilized oligonucleotides [66], inorganic silica [67], etc. The capacity value for synthetic DNA is experimentally validated and visually reported in Fig. 2. In this instance, the molecular storage unit is dehydrated synthetic DNA pool.

Significant efforts are being made to use inorganic molecules for data storage. Current efforts are based on the use of four-color printing of clusters to reveal their extreme nonlinear optical properties. These properties should be distinguishable enough for a spectrometer to read them and thus capture the information they contain. The fundamentals of such molecular clusters are described in several works [87], [88], [89].

Another aspect of molecular storage is organism-based. This corresponds to maintaining certain ***cell cultures***. It effectively relies on integrating short synthetic DNA strands (i.e., oligonucleotides) into a biological organism *in vivo*. In turn, the organisms can store and duplicate the information on an *ad hoc* basis. This special type of medium was researched to insert synthetic DNA fragments encoding data and inserting them into the genome of bacteria, fungi, and plants. This was successfully accomplished in *Escherichia coli*, *Bacillus subtilis*, *Pichia pastoris*, and *Arabidopsis thaliana* [71], [72], [73]. Estimates for a single gram of bacteria indicated an advertised storage capacity of more than 900 TB. However, an experimentally validated approach confirms using bacterial cell cultures with 445 KB of digital files in synthetic DNA [73].

***Synthetic metabolomes*** are another promising molecular medium. Metabolomes comprise the complete set of small molecules found in a biological system. Unlike DNA and protein molecules, they are small in mass, abundant, and more structurally and energetically diverse [76]. The synthetic metabolomes consist of a mixture of metabolites (e.g., Galactose, Tryptophan, etc). These are spatially arrayed in thousands of nanoliter volumes on a physical multi-well array or plate. In turn, each resulting volume contains a prescribed mixture from a library of purified metabolites, i.e., a synthetic metabolome. This approach demonstrated the storage of many image data. The largest is a 17,424-bit image requiring approximately 70 KB. This image was written into 1,452 mixtures from a 12-metabolite subset of the library [76].

Another noteworthy addition concerns non-genomic molecular media. They have also been demonstrated yet have not been included in this study due to their experimental nature. One type of such media relies on fluorescent dyes on polymer films or the rotaxane molecular architecture [90], [91]. Another type relies on creating nano-structures to obtain an etched crystalline quartz or even a thin diamond layer [92], [93].

### 2.4 Industry-based UI and visualizations

From the early days of the computer, the amount of used information in the storage devices was important. Early devices showed the usage of the storage medium in percent or only the occupied space next to the capacity of the medium or both. This information was presented in a textual form and a human-readable format. This refers to encoding the information in ASCII or Unicode text rather than binary data. Many technological breakthroughs, such as nanoscale circuits, enabled more compact and reliable solutions with a higher element density. Various sensors were introduced to different storage types, for example, temperature sensors. This led to the monitoring of meta-information, which in turn allowed the prediction of the device lifespan. In turn, UIs were upgraded with further characteristics such as Temperature or Speed of the storage device. The advancement in graphical processing power also opened up further possibilities. First and foremost, it enabled the visualization of the available information for the capacity property. Later, it was used to visualize partitioning of the storage medium, file structure, usage, etc. We divide our investigation into early, pre-modern, and modern operating systems.

**Fig. 3:**
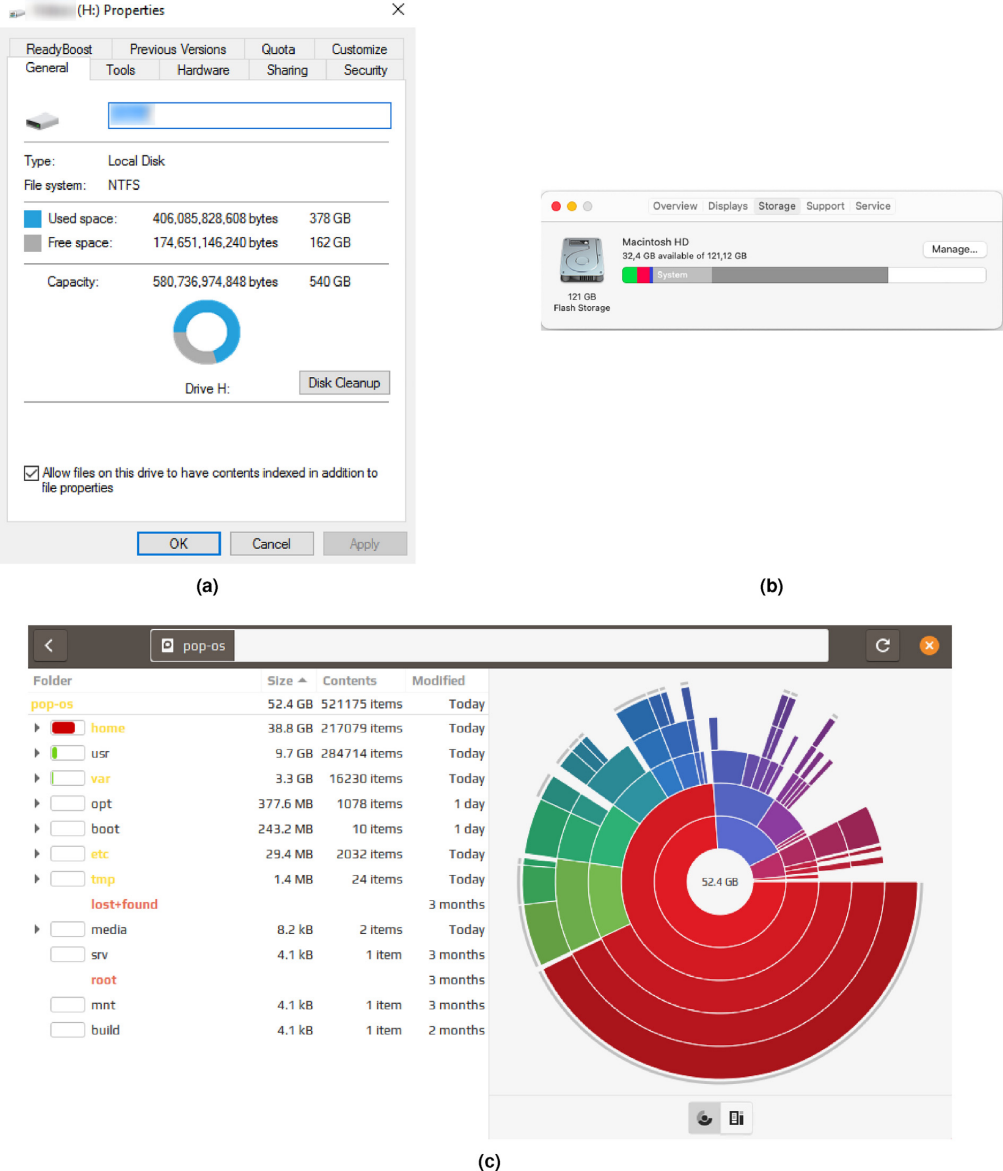
*Screenshots of data usage visualizations for different Operating Systems.* Part-to-a-whole visualization variations are used. (a) Donut chart representing the local disk usage on Windows 10. (b) Stacked bar chart (horizontal) representing the amount of memory used by different file types on Mac OS X Catalina. In the most recent version, Big Sur, only used space is shown in relation to the available space. (c) Sunburst diagram representing the amount of memory used by different directories on Linux (Pop!_OS 20.10).

Early systems introduced built-in tools that report detailed information about the user’s storage devices. These systems comprised the report of textual information for different properties, yet mainly focused on capacity [94]. Such systems align with the pre-modern arrival of the personal computer with Windows 95${}^{®}$ or System 7${}^{®}$, and their adoption until Windows XP${}^{®}$ and Mac OS X${}^{®}$. The capacity of a storage device was one of the most important properties and was often visualized as a horizontal stacked bar chart displaying used and free storage space. Modern systems have built-in tools and visualize storage capacity with pie charts and some of its variants, like doughnut charts, or even multi-level pie charts, as seen in Fig. 3.

**Fig. 4:**
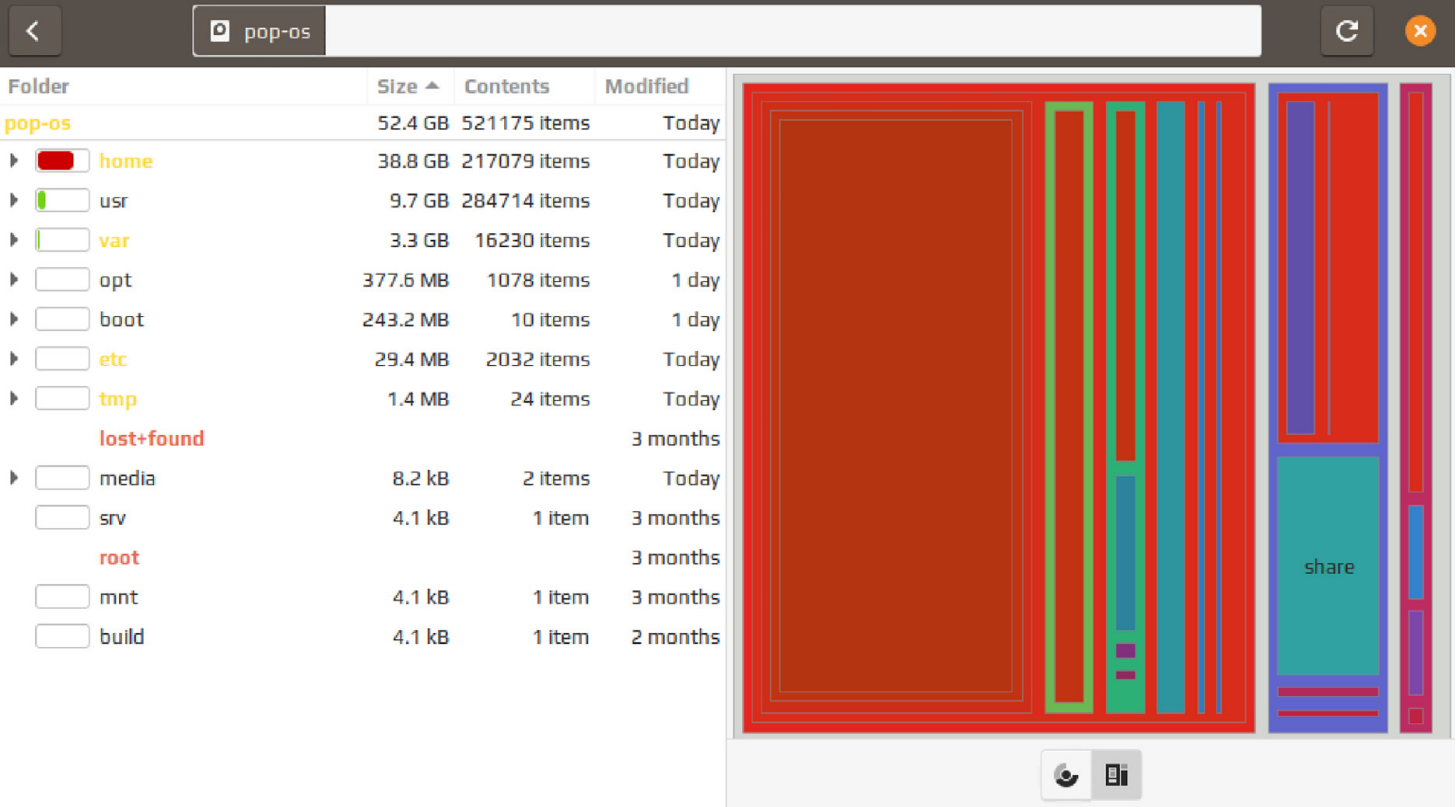
*Alternative visualisation on a Linux distribution.* Tree map chart representing the amount of memory used by different directories on Linux (Pop!_OS 20.10).

**Fig. 5:**
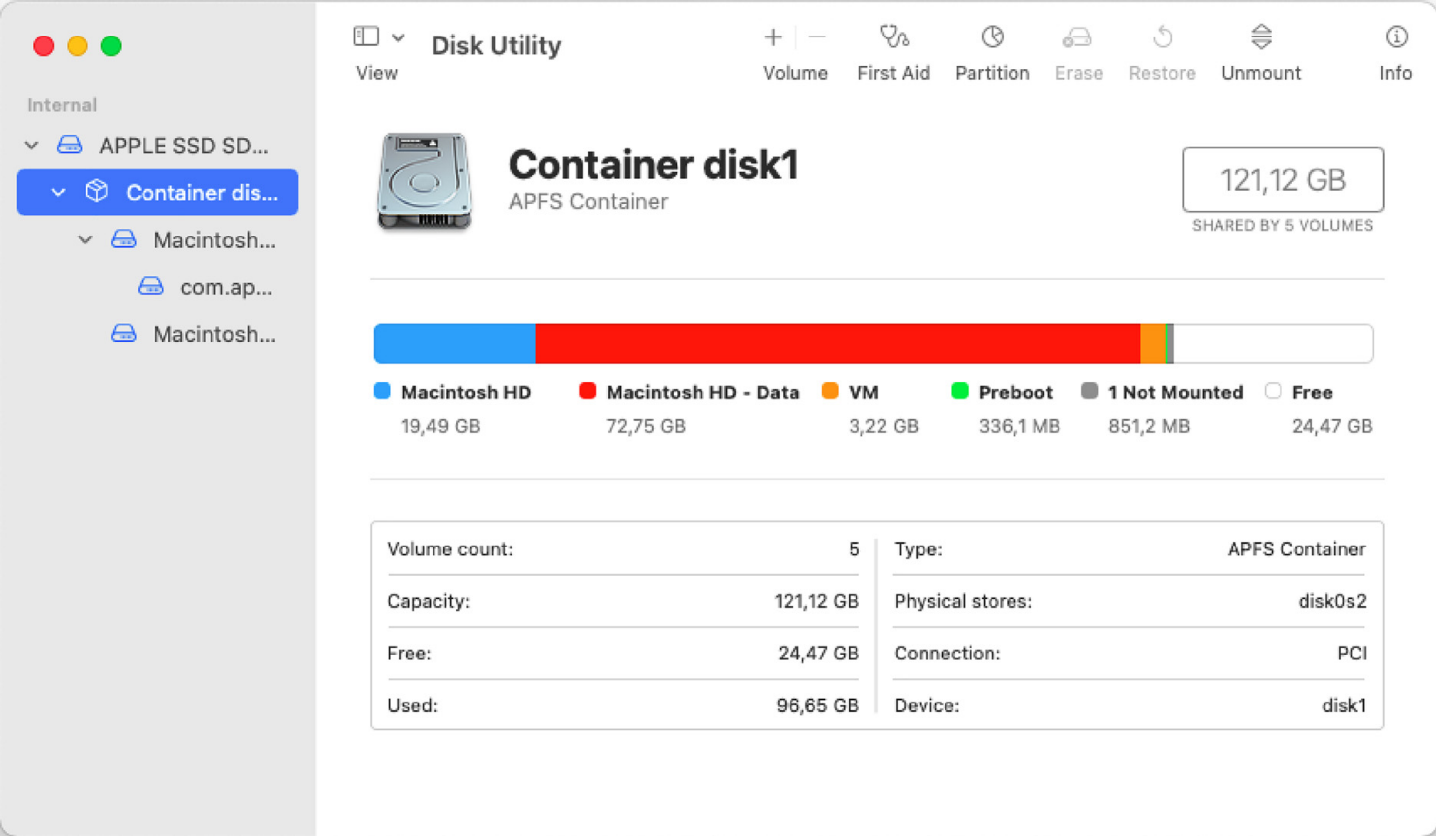
*Additional information on Mac OS X.* Supplementary data of how the storage device is partitioned and other important information such as the type of physical connection and the name of the disk are reported in textual form and tabular format on Mac OS X.

Along with previously mentioned visualization methods, certain OS offered a built-in representation of used storage space in a tree map chart, as seen in Fig. 4. In most cases, the visualization of usage presents an overview first, details second. Generally, the overview is the stacked horizontal bar chart, while details may be observed in visual and textual form simultaneously. Furthermore, supplementary storage device information, for example, SSD temperature or mount point, is presented in textual form. Fig. 5 depicts this occurrence.

### 2.5 Survey results

As stated earlier, the survey was conducted on two different groups: (a) domain experts in molecular data storage, and (b) the general public. Nineteen responses were collected from the expert group and thirty from the general public.

In the first group, 68.4% of participants identified themselves as male, and 31.6% as female. Exactly 47.4% put computer science as their field of research, followed by biology at 36.8%, then chemistry at 15.8%, then physics and mathematics at 5.3%. Nearly 60% reported having 10 or more years of experience in their respective field, followed by 31.6% who have 0 to 3 years of experience, and the rest having 3 to 5 years of experience. When ranking the TOP 3 properties, and while considering themselves domain experts, participants chose the following in order of importance: 1. lifespan, 2. capacity, and 3. accessibility. These results changed when considering themselves as members of the general public. The properties of accessibility and lifespan switched places, placing accessibility first and lifespan last. All TOP 3 ranking results are also presented in Table 2.

**Table 2:** *Survey results.* Cells with the highest votes are highlighted. For the expert pool, the TOP 3 properties are lifespan, capacity, and accessibility. When experts are asked about their opinion as members of the public, the order of the TOP 3 changes to: accessibility, capacity, then lifespan. For the general public, the results are the same as when the expert pool stated their opinion as members of the general public.

Exactly 43.3% of the participants belonging to the general public group identified themselves as male, followed by 50% identified as female, while 6.7% preferred not to state their gender. As for the field of study, 60% reported computer science, 16.7% social science, 13.3% biology, 10% medicine, 6.7% mathematics and the rest was split between economics, and business informatics. The general public was not asked the question about their experience. The results gathered from this group, i.e., the general public, are reported in Table 2. In this case, the TOP 3 was: 1. accessibility, 2. capacity, and 3. lifespan.

To support the visualization of the TOP 3 data storage properties, we designed and implemented a User Interface (UI) adapted to both audiences. The methodology for property-based visualizations and the UI is described in the Methods section. The implementation of the UI and the source code used to create all of the figures are uploaded as part of the supplementary material and are also available at the *TVSDS* GitHub repository:  https://github.com/AAnzel/TVSDS. The survey and its results are provided with this manuscript as part of the supplementary material.

## 3 Methods

The literature search was possible thanks to an array of different scholarly literature databases: Google Scholar, Europe PMC, IEEE Xplore. While the experimental part of the paper consisted of generating a survey, analyzing results, and creating an adequate UI and data visualizations.

### 3.1 Literature search

Relevant search keywords were used for different parts of this paper. A non-exhaustive list of used keywords is: data storage, novel media, storage device, storage medium, DNA storage, storage visualization, molecular medium, molecular storage. Apart from the literature search, our paper also includes knowledge gathered from several archives, for instance, the IBM Archives, The Internet Archive, and the Museum of Obsolete Media. For certain data storage technologies, like HDDs, HVDs, SSDs, we also incorporated relevant facts stated in their storage standards, that are publicly available online.

### 3.2 Ranking survey

For the survey, we used Google Forms and presented it as follows. First, a brief explanation of the survey was given followed by a one-sentence description of each data storage property: accessibility, capacity, lifespan, mutability, and usage. Second, participants were asked to rank them in a TOP 3 fashion. The expert group was asked to rank properties as experts in the molecular data storage domain and as members of the general public. Third and last, demographics data was collected. We gathered the results of the survey and proposed a new UI for the property-based visualizations. The survey results laid out in Table 2 are divided into three groups: (a) the domain expert opinion, (b) their view as a public, and (c) the opinion of the general public.

### 3.3 User Interface

**Fig. 6:**
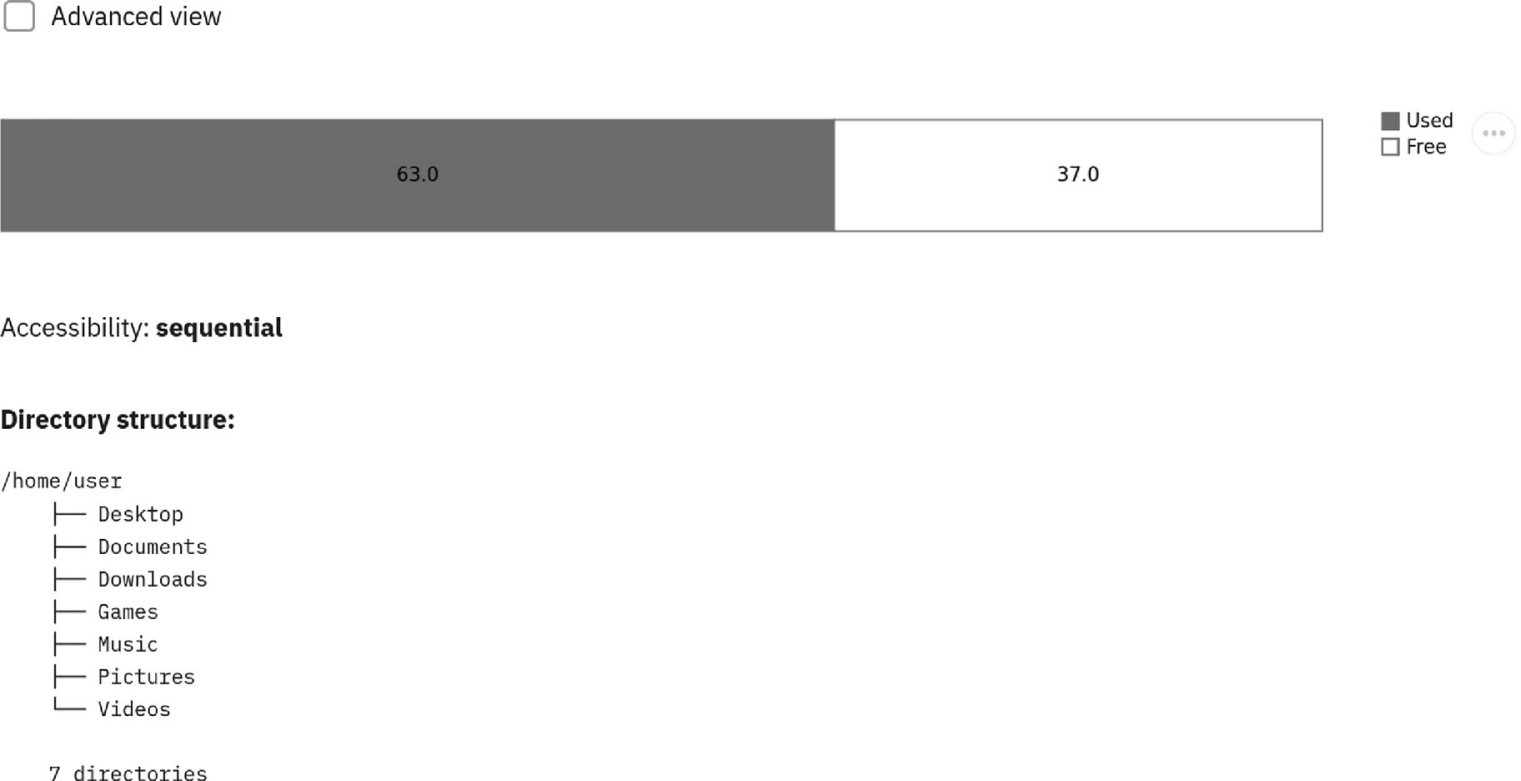
*The basic view.* It provides an overview of the used versus the free storage space as well as the main properties that are required for the general public.

By coupling the results from the survey, the literature search, and the visualization standards to display data storage information, we propose an adapted user interface and state-of-the-art visualizations. The proposed User Interface (UI) consists of two views: the basic view to suit the needs of the general public, and the advanced view for domain experts. The default home view is the basic view. It serves as an overview by presenting the accessibility (sequential versus random), the usage or how much of the media is already in use (in percentage), and the capacity of the media in kilobytes (KB). In addition, a file hierarchy is presented in textual format. The basic view component of the UI can be seen in Fig. 6.

**Fig. 7:**
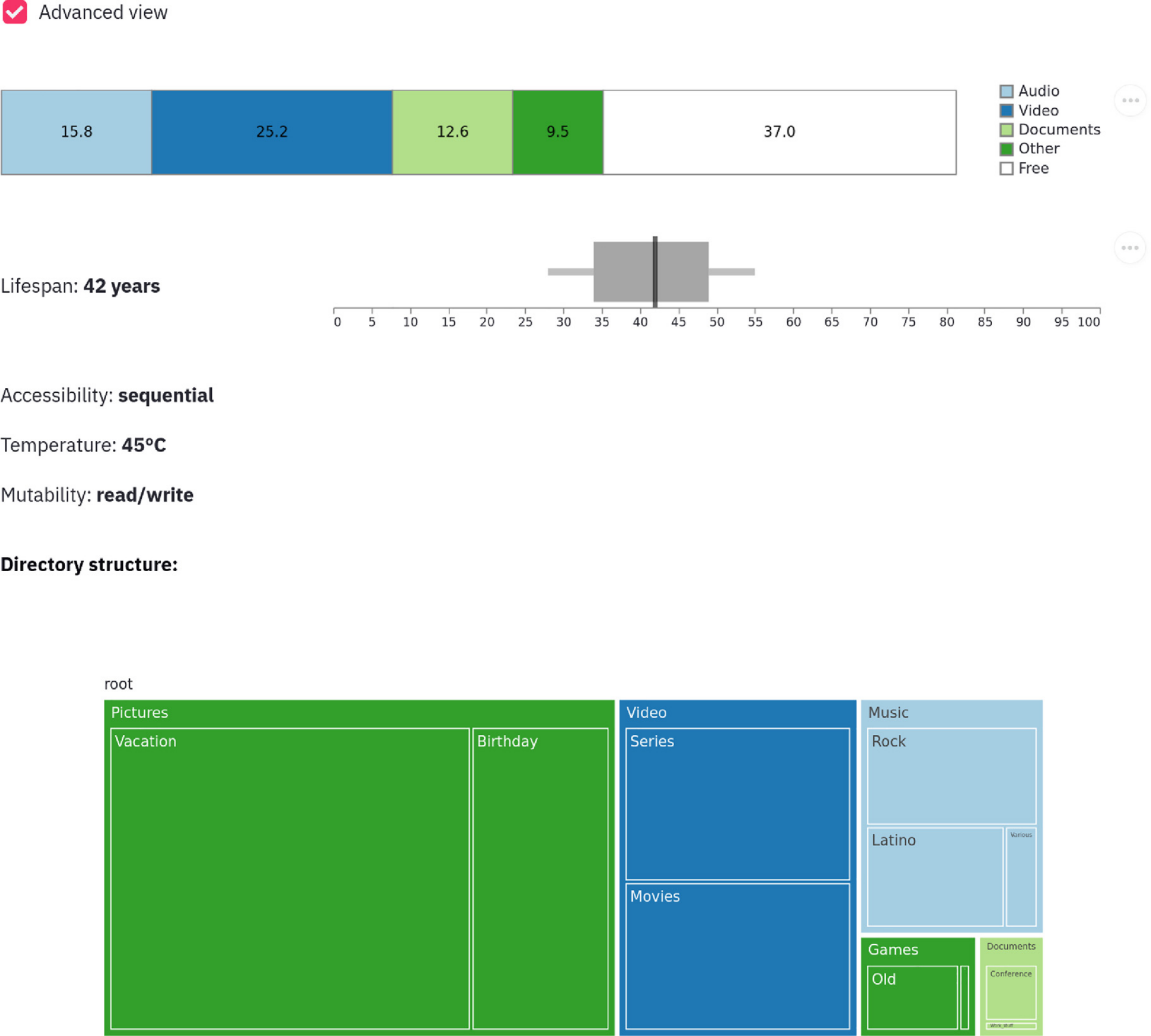
*The advanced view.* If the *Advanced view* checkbox is ticked, the storage medium properties are displayed in order of importance for the expert audience.

**Fig. 8:**
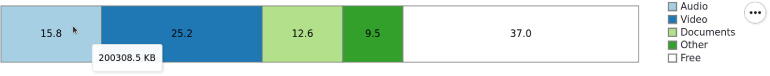
*Capacity/usage tooltip in the advanced view.* Supplementary information is shown while mouse-hovering over stacks.

**Fig. 9:**
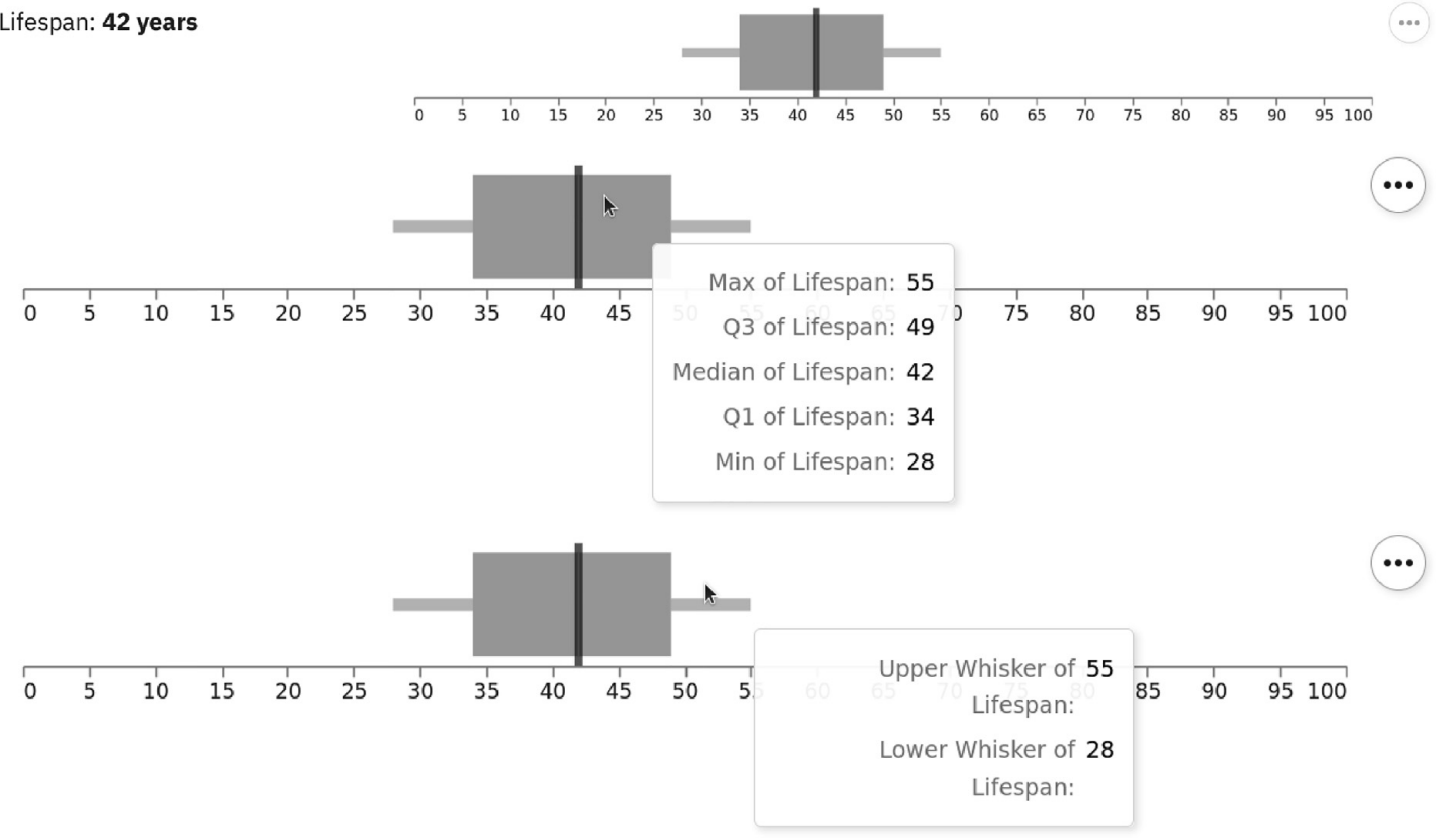
*Lifespan visualization.* The lifespan property is presented with textual and visual information. Detailed information of lifespan estimation is presented in an overlay window while hovering over the gray area, as well as over the whiskers.

The advanced view can be toggled by a select box button to display further details. It specifically includes additional properties (e.g., lifespan) and further details for the usage and the file hierarchy. This is shown in Fig. 7. The advanced view benefits from mouse hover and mouse click events to provide granular details for each visualization. For example, the mouse hover event for the capacity/usage visualization reports the actual size in kilobytes (KB) of a specific file type category (e.g., audio files), as seen in Fig. 8. The mouse hover event for lifespan visualization gives detailed information on lifespan estimation, as shown in Fig. 9.

**Fig. 10:**
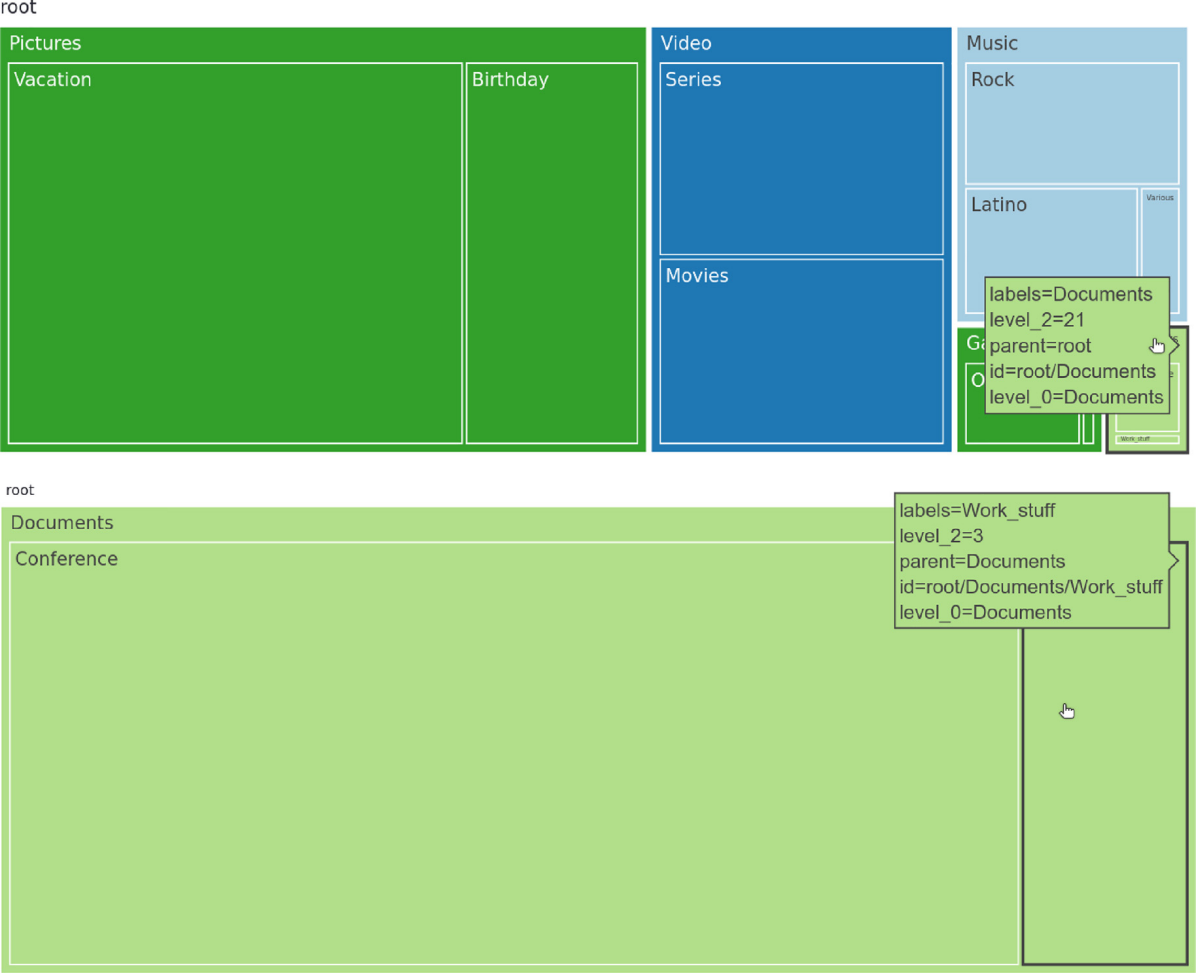
*Directory hierarchy visualized with a tree map chart.* Each rectangle is colored according to the file type category present in the corresponding directory. In the image below, the *Documents* folder is now a top-level directory, with root as its parent directory. Supplementary information is shown while mouse-hovering over rectangles.

On the other hand, selecting a directory by a mouse click within the directory hierarchy visualization makes the directory expand to occupy the whole pixel space of the visualization. The chosen directory is now considered a top-level directory, and the visualization shows the hierarchy of its subdirectories, with all previously top-level directories laid on top of the visualization. This is shown in Fig. 10. The UI reports the properties by relying on the TOP 3 ranking results, as seen in Table 2. In light of the survey results, the order of appearance of the visualizations is adjusted to fit the reported importance of the studied properties depending on the audience, and is updated accordingly in its corresponding view (basic vs. advanced). The visualizations present in both UI views dynamically adapt to the current page width, maximizing the ink-to-pixel ratio.

### 3.4 Property-based visualizations

By considering the state-of-the-art data visualizations for data storage, we propose 3 main visualizations for the ranked properties: usage, capacity, and lifespan. We follow the nested model of visualization to describe the visual encoding [95]. The properties are encoded as follows: capacity as a real number, while usage is reported as a real number and in percentage (%), lifespan as an integer, and accessibility uses the boolean data type (0 or 1). Textual information is represented as UTF-8 text strings of variable lengths.

First, for both usage and capacity, a horizontal bar chart is employed. Bar charts are very effective at displaying part of a whole, and visually comparing metric values across different subgroups of the data at hand. The basic view shows only two parts or two stacks, free and used space. While the advanced view details which kind of file type categories (i.e., audio, video, documents, other) occupy the storage space, and by how much (in percentage and in KB). Except for the free space stack, each stack is visually encoded using the area and color channels. The area channel represents the used space: each part as a percentage to a whole (i.e., maximum storage capacity in KB). The color channel relies on the categorical or nominal encoding of each file type category. Four file types are considered excluding free space: audio, video, documents, and other are mapped to colorblind-safe categorical colors: #A6CEE3, #1F78B4, #B2DF8A, and #33A02C, respectively. The encoding of the color channel follows the state-of-the-art rules to colorize a data visualization [96]. Moreover, each stack benefits from textual overlays to report the used space in percentage (%). This is presented in Fig. 8.

Second, we used a whisker chart for the lifespan property. It combines a bar and whiskers as well as a vertical line. The latter spans the chart height and encodes the quantitative property (i.e., estimated lifespan) of the medium by relying on the position channel. The estimation of the lifespan is encoded using the area (bar) and the position (line). Since the lifespan of a medium largely depends on various external factors, the whiskers represent the confidence interval of this estimate, while the 95% interval is represented as a gray area. The lifespan property is also reported in a textual form as seen in Fig. 9: the estimated lifespan is 42 years.

Third and last, and as integrated into different operating systems, we implemented a directory hierarchy visualization by using the tree map visualization. The rationale of this visualization method is to maximize the pixel space at our disposal. It is highly efficient since it uses a space-filling technique and relies on creating multiple rectangular areas [97]. Each directory represents a rectangle, where hierarchies between different directories are encoded with containments to create a nested layout. A rectangle is encoded by its area and color channels. The larger the area, the larger the rectangle, the larger the directory. The color channel follows the aforementioned nominal encoding in the usage/capacity chart with 4 file type categories. The name of a directory is encoded in textual form, with each name contained in a rectangle representing that directory, as seen in Fig. 10. Additionally, the accessibility property is not visualized but reported in a textual manner. This can be seen in Fig. 7. The implementation is done using Python 3.9, Altair 4.1, Plotly 4.14.3, and Streamlit 0.82.0 [98], [99], [100], [101].

## 4 Conclusion

Existing storage technologies are insufficient for the long-term storage of the large amounts of data generated in all areas of life. Novel and alternative media propose to extend the existing storage capacity with molecular storage media and devices. Hence, also reducing the risks for information loss in long-term data storage. Although technological advances are being made for molecular storage media types, there has been a lack of efforts for standardization. This is especially relevant before the adoption of upcoming storage media, such as synthetic DNA. Indeed, standards not only improve ways to standardize information storage for long-term archiving (i.e., lifespan), but also help researchers meet certain criteria when developing novel storage media. It is also important to consider the ways we digest the information that is conveyed by storage media properties. By means of ranking, our survey permitted us to identify relevant properties for domain experts and members of the public. Moreover, thanks to an analysis of the industry-based UI and visualizations, we were able to observe and converge to specific design choices. For the bar chart, the use of the color channel was mapped to file type categories (audio, video, documents, and other), while the area channel depicts the used amount of the available capacity (often in percentage or in GB). On one hand, the horizontal stacked bar chart had been a preponderant choice for the display of the usage of a medium’s capacity. On the other hand, the tree map had also been a common choice to display file directories and file hierarchies. For the tree map chart, the color channel encodes file type categories, and the area channel encodes the amount used by said files. In addition, the containment provided by this space-filling visualization method helped lay out hierarchies among different directories. Owing to these industry-based and widely accepted charts, we developed a user-settable approach to toggle between an overview and details. This corresponded to the basic vs. advanced view. The latter provided additional data, including further annotations, and unraveled further details about the storage medium.

## 5 Discussion

First, and thanks to our survey, we were able to separate the different storage properties by relevance. Besides this, we found that the TOP 3 properties converged for the general public and when the expert participants considered themselves as members of the general public.

Second, by having the survey results, we were able to propose a new UI that is generalizable and could also be specifically used for molecular storage media or upcoming novel media. Even though accessibility was chosen as the most important data storage property, it might be reasonable to shift its position to second place. The rationale is that visual information is more relevant and has greater power. From a historical point of view, it is more reasonable to present information rather visually than textually. That is to say, a user should be first confronted with some type of graphical or visual representation. That is why we first present a user with the capacity/usage visualization first, then we present the other properties as ranked by the survey results.

Third, the need for standards extends beyond the currently provided example. Indeed, as seen in the proposed UI and visualization, certain properties have an intrinsic uncertainty. This may either be due to the fact that the property relies on estimation, or that a property is measured. In the general case of the lifespan property, estimates vary to include multiple storage and usage conditions such as temperature, number of read/write, recycling of the media, mechanical movements, etc. We argue that estimates could benefit from standardization so that precise values with certain confidence intervals may be reported. In this regard, the literature lacks evidence-based estimates. In the example case of the lifespan property of synthetic DNA, the theoretical limit is supported by the oldest known preserved DNA in existence, aging approximately one million years [102]. Moreover, the experimentally validated capacity for synthetic DNA seems small in relation to the theoretical limit. This limit is estimated to reach multiple orders of magnitude higher than the presented capacity value. However, the current costs of storing data inside a DNA molecule greatly limits the experimental validation of this maximum. Furthermore, methods and algorithms developed in the field of genomics may benefit current data storage approaches using DNA. This includes, but is not limited to, data compression and indexing [103], [104], [105]. In the example case of a synthetic metabolome, the capacity property depends on the number of metabolites present in a metabolome. That is to say, a measurement is made. Such aspects of uncertainty and the way such information is presented for general consumption remain an open question.

Fourth, a noteworthy example of a novel and experimental storage medium is the Data Sticky. Although very promising we excluded it from our findings of the literature search. Data stickies are inspired by sticky notes, such as Post-Its${}^{®}$. A realistic implementation exists, namely Post-Bit, where a small e-paper device stores multimedia contents and allows for paper-like manipulations [106]. They combine the affordability of physical tiny sticky memos, the digital handling, and the display of information using electron ink or e-ink [107]. Once this ink settles into an image, the display reflects light just like ordinary paper; as a non-volatile medium. Recent advancements in nanotechnology proposed a larger storage capacity using graphene paper and e-ink [108]. Estimates put such upgraded data stickies between 4 and 32 GB. There have also been considerable efforts to create nanoscale data storage using graphene. A promising strategy addressed high precision writing and drawing on graphene nanosheets by manipulating electrons with a one nanometer-based probe [109].

Fifth and last, even though our UI proposal is applicable to molecular data storage media, more visualization research is warranted for medium-specific properties. Our work herein reported properties that are in the broadest sense shared and relevant. Since molecular data storage media is in active development and research, we can expect some new storage medium-specific properties that could be more important than the properties we reported. That means that UIs and visualizations should evolve and adapt to the new storage media types and all of the important, specific properties those media types may have.

## Author contributions statement

A.A. curated the data, implemented the survey, validated the results, and created the visualizations. A.A., D.H., and G.H. wrote the original draft. G.H. supervised the work. All authors reviewed the manuscript.

## Declaration of Competing Interest

The authors declare that they have no known competing financial interests or personal relationships that could have appeared to influence the work reported in this paper.
